# Importance of work-related factors for post-retirement labour market participation

**DOI:** 10.1371/journal.pone.0347152

**Published:** 2026-04-15

**Authors:** Anu Polvinen, Sanna Tenhunen

**Affiliations:** Finnish Centre for Pensions, Helsinki, Finland; University of Verona: Universita degli Studi di Verona, ITALY

## Abstract

Today’s pensioners are healthier than ever, and many participate in the labour market after retirement. Some pensioners continue to work for financial reasons, but in most cases the motives for work are other than financial. Many sociodemographic factors are associated with post-retirement employment, but less is known about the role of work-related factors. This study examines the relationship between work-related factors and post-retirement employment among individuals who have recently retired on an old-age pension. The study is based on a Finnish survey conducted in 2022 (response rate 68%), supplemented by register data on 3,196 individuals aged 63 and over who retired from paid employment with an old-age pension between 2019 and 2021. Logistic regression models were used to estimate how work-related factors are associated with post-retirement employment. Information on work-related factors pertains to the last job held before retirement. Higher job demands and lower social support were associated with a lower likelihood of working in retirement. A higher physical workload was associated with a higher likelihood of working in retirement, especially among those working for financial reasons. Shift work, part-time work, and temporary work before retirement were also associated with a higher likelihood of working. Managers and professionals were more likely to work in retirement than lower-grade non-manual workers. The findings of this study highlight the importance of work-related factors in post-retirement employment decisions. Policies aimed at promoting such employment should therefore focus on improving working conditions before retirement. Furthermore, since work-related factors are differently related to financial and non-financial retirement motives, policy frameworks should move beyond one-size-fits-all solutions.

## Introduction

In recent years, working while receiving an old-age pension (or ‘working in retirement’, ‘working after retirement’, ‘post-retirement employment’) has become increasingly common in Western countries [1–2]. Old-age pensioners today generally have higher levels of education and better health than previous generations, and many express an interest in continuing to work alongside their pension [3–6]. Retired people are a valuable resource in the labour market: they have much experience and are often interested in working and willing to consider working flexible hours. Research has shown that favourable working conditions encourage people to stay in work longer [7–12]. However, little is known about how work-related factors are associated with working while receiving an old-age pension. This association may be different among those retirees who work for financial reasons and those whose motives are non-financial.

Retirement does not always mean a complete and immediate transition from work to full retirement [13–17]. People reaching retirement age may choose to continue working rather than retire, to retire full-time rather than work, or to continue working full-time or part-time while receiving a pension. Previous literature has shown that many people continue to work part-time in various ‘bridge jobs’ en route to full retirement, suggesting that retirement is often a gradual process rather than an abrupt change [14,17,18]. Working in retirement can also be understood from a life course perspective and through continuity theory, which suggest that individuals seek to maintain their identity, lifestyle, and social roles throughout their lives, including during the transition from work to retirement [19–25]. As post-retirement employment continues to expand, the motives and factors behind decision-making on post-retirement employment provide an interesting area for research.

Participation in the labour market at an older age is more common when physical working conditions and psychosocial circumstances are favourable. Many work-related factors, such as type of work, physical and psychosocial working conditions, and the possibility of flexible working hours, are important in the decision to continue to work beyond the traditional retirement age [8,10–12,26,27]. A previous study [26] suggests that older workers who have control over their work tasks, who are given opportunities for learning and using their skills, and who are rewarded and acknowledged for their achievements, remain at work for longer. While earlier research highlights the importance of favourable working conditions and good physical and mental health in continuing at work [8,10–12,26,27], most of the research focus has been on extending employment beyond retirement age rather than on working while receiving a pension. This area remains underresearched.

A previous study on working retirees found that the majority experience favourable working conditions [28]. Working retirees tend to work in low-strain jobs, which are characterised by part-time work, low physical and mental demands, and a high level of job control. However, a smaller proportion participate in high-strain jobs that are characterised by what are considered undesirable working conditions, such as full-time work, high physical and mental job demands, and a low level of job control [28]. Furthermore, previous studies have shown that working in retirement is more common among those with higher educational level, socioeconomic status, or occupational position compared to those with lower levels [6,29–32]. Typically, individuals in higher-level occupations experience less physically demanding work and have greater opportunities to influence their working conditions, for example.

In addition, previous working arrangements and career length may be associated with continuing to work in retirement. Individuals who have spent a greater proportion of their careers in part-time work or self-employment, as well as those with flexible careers throughout the life course, are more likely to remain in employment after retirement [24]. This is possibly because these individuals are accustomed to working in various roles, modifying their job tasks, or changing employers, meaning they are better placed to continue working in retirement. Previous studies [33,34] have indicated that part-time work or short careers may be associated with continuing to work in retirement mainly for financial reasons, for example in response to low levels of pension accrual or financial well-being.

Financial needs have been found to be an important motive for working beyond the age of 65 [7,35]. Therefore, low earnings over the career course may be one reason why some people decide to continue working while receiving a pension. Some studies have reported a U-shaped association between income and working in retirement, with both those with the lowest and those with the highest incomes often staying on at work [36,37]. People working on low wages may have a financial need to work, while those with higher incomes are more likely to be motivated by non-financial reasons such as maintaining one’s social network, maintaining a professional identity and good health, having an appropriate balance of activities, or being able to use one’s job-related skills [7,35,38,39]. Working in retirement offers an opportunity to maintain social relationships and professional identity and to avoid the negative effects of retirement. However, for those working for financial reasons, this may not necessarily be the case.

Extending working lives is an important social objective in ageing Western societies that are facing labour shortages and public finance challenges and struggling to ensure the sustainability of their pension systems. Population ageing and labour shortages highlight the need to make the best possible use of pensioners’ skills in the labour market. Allowing pensioners to work while receiving a pension can help to strengthen the financial base of the pension system and reduce pressure on public finances. Post-retirement employment provides a flexible transition to retirement, promoting the well-being of pensioners and encouraging active ageing. Working in retirement can also have significant economic and social impacts, as it improves an individual's financial position, increases government tax revenues, strengthens social relationships, and potentially reduces the need for social and health services. The nature of work and working conditions are crucial to whether old-age pensioners can continue to work: lighter, flexible, and part-time jobs may facilitate continued employment, whereas physically and mentally demanding jobs may prevent it. It is therefore essential to study these factors in more depth.

Finland is an example of a Western society with an ageing population and a comprehensive public pension system that allows for a flexible transition from work to retirement. The importance of work-related factors in decisions on post-retirement employment in the Finnish context likely provides a useful point of comparison for other countries aiming to extend people’s working lives during retirement.

The novelty of our study lies in its access to extensive survey data on recent retirees from paid work, focusing on their experiences and perceptions of pre-retirement work. Our first aim is to examine how several work-related factors such as physical workload, psychosocial working conditions, work arrangements, occupational class, and length of working career are associated with post-retirement employment. Furthermore, we assume that these associations may differ between those who continue to work for financial reasons and those whose work in retirement is driven by other motivations. Thus, the second aim of our study is to explore whether these factors are differently associated with post-retirement employment in these two groups. From a societal perspective, it is important to better understand these relationships as working lives continue to get longer.

### The Finnish pension system and working in retirement

Finland has a flexible retirement age, giving people the option to retire any time after reaching the minimum age for claiming an old-age pension [40]. In connection with the 2017 pension reform, the decision was made to incrementally raise this minimum age from 63 to 65. There are exceptions, though, as some public sector employees have a lower professional retirement age.

People who decide to continue working after retirement are required to terminate their existing employment contract on retirement and sign a new one. There are no limits to how much people can earn while drawing an old-age pension. Working retirees will continue to earn new pension entitlements up to the age when the insurance obligation ends [41]. Typically, retirees who continue working after retirement return to the workforce relatively soon, often in part-time or occasional roles, and many remain in the same occupations or positions they held prior to retiring [6]. In Finland, almost one in five pensioners aged 65–80 work in retirement [42]. This figure has been found to be higher among those who have recently retired from paid employment and among the self-employed [6,42].

## Materials and methods

The study is based on the ‘From work to retirement’ survey collected by the Finnish Centre for Pensions at the end of 2022. The survey was sent to 5,000 randomly selected pensioners who had retired from paid employment with an old-age pension between 2019 and 2021. It included several questions about retirement and post-retirement work. The survey data was linked to Finnish Centre for Pensions and Statistics Finland register data. The response rate was high at 68 per cent.

The analysis was restricted to old-age pensioners who were at least 63 years when they retired. The final study population included 3,196 respondents who had been retired for approximately one to three years at the end of 2022.

The study was conducted in accordance with national data protection laws and the guidelines of the Finnish National Board on Research Integrity [43]. At the Finnish Centre for Pensions, data processing procedures comply with the EU General Data Protection Regulations and Finnish national legislation [44,45]. Persons processing the data are subject to statutory secrecy provisions. The survey participants were informed about these privacy measures. Due to legal restrictions and data protection regulations, the datasets cannot be shared publicly.

### Post-retirement employment

The dependent variable was based on a question asking whether the respondent was or had been working while receiving an old-age pension (yes/no). The question did not limit the amount of earnings from work or the length of employment. One-third (33%) of the respondents said that they had worked while receiving an old-age pension.

### Motives for post-retirement employment

The survey included a question about financial motives for working in retirement. This question was only asked of those who said they had worked in retirement. The financial need to work was defined by the following statement: ‘I want to work because I am experiencing financial difficulties’. The response options were 1=’fully agree’, 2 = ’partly agree’, 3 = ’don’t know’, 4 = ’partly disagree’, and 5 = ’fully disagree’. The responses ‘fully agree’ and ‘partly agree’ were considered to represent a financial need to work.

### Job characteristics

Information on pre-retirement work characteristics was collected by asking respondents about their views on various aspects of their work. Physical workload was measured by the statement ‘My job was physically too demanding’. Psychosocial working conditions were measured by several statements describing job demands, job control, and social support at work. There were three job demand statements: ‘My work was too busy or there was too much work’, ‘The job’s skill requirements increased too much’, and ‘There were too many changes in my job description or in the organisation’ (Cronbach’s alpha 0.71). Job control refers to the individual’s ability to influence their work environment, and that was measured with the statement ‘I was able to influence my work’. Social support for older employees was measured by two statements: ‘Older employees were appreciated at my workplace’ and ‘Older employees were supported to continue working until their old-age retirement age’ (Cronbach’s alpha 0.73).

For all questions the response options were 1 = ’fully disagree’, 2 = ’partly disagree’, 3 = ’don’t know’, 4 = ’ partly agree’, 5 = ’fully agree’. In each case, the sum variables were created by dividing the sum of the responses by the number of statements so that the distribution of scores ranged from 1 to 5, with a higher score indicating higher job demands and physical workload, more job control, and more social support.

The questions regarding pre-retirement working arrangements elicited information on whether respondents had engaged in shift work or part-time work and on the permanency of the employment contract. The shift work item also elicited information on whether the pre-retirement job had involved working in two or three shifts, including night work. We also asked whether the pre-retirement job had been full-time or part-time. Type of employment was classified based on whether the pre-retirement employment contract had been permanent or temporary (or other).

### Occupational position

Information on pre-retirement occupational position was derived from register data and based on the International Standard Classification of Occupations (ISCO-8). We combined some groups and labelled them as ‘Managers and professionals’ (groups 1 and 2), ‘Lower grade non-manual workers’ (groups 3 and 4), ‘Skilled manual workers’ (groups 5–8), and ‘Elementary workers’ (group 9).

### Length of working careers

Career length was derived from register data, based on records of pension-insured employment either as an employee or as self-employed from age 18 until the end of 2022, when the survey was conducted. Three categories of career length were distinguished: careers lasting less than 35 years before old-age retirement (short career), careers lasting 35–45 years (medium-length career), and careers spanning 45 years or more before retirement (long career).

### Self-rated health and ability to work

Self-rated health was measured on a five-point Likert scale ranging from good to poor. For analysis, it was divided into three categories: good, moderate, and poor. We also included the respondents’ own assessments of their ability to work relative to their career-best ability to work, with scores ranging from 0 to 10, 0 meaning ‘completely unable to work’ and 10 meaning ‘highest ability to work’. We classified ability to work as excellent (10), good (8–9), medium (6–7), and poor (0–5).

### Statistical analysis

The association between work-related factors and post-retirement employment was assessed using SAS Enterprise Guide 8.2. Logistic regression analyses estimated how work-related factors (physical workload, psychosocial working conditions, work arrangements, occupational class, and length of working career) were associated with working in retirement. The models were adjusted for age, gender, self-rated health, and ability to work. Additionally, a multinomial logistic regression model was used to explore those working in retirement for financial reasons and those motivated by other factors. The reference group consisted of individuals not working while receiving a pension. Odds ratios and their 95% confidence intervals were calculated.

## Results

One-third (33%; N = 1,032) of the study population reported that they had worked while receiving an old-age pension. Of these, 36 per cent reported that they had worked out of financial need. The majority of those who worked in retirement (64%) felt that they had no financial need to work.

Table 1 shows the descriptive statistics for work-related factors (such as physical workload, psychosocial working conditions, work arrangements, occupational position, length of working career), self-rated health, work ability, and age by post-retirement working status. Work-related factors differed between those who had worked in retirement and those who had not. These two groups were almost equally likely to find their work physically demanding. However, those who worked for financial reasons were more likely to feel their work was excessively physically demanding (mean = 2.34; 95% CI (2.19–2.49)) than those who worked for non-financial reasons (mean = 1.81; 95% CI (1.72–1.90)).

**Table 1:** Descriptive statistics for work-related factors, self-rated health, ability to work, gender and age by post-retirement work status.

	Post-retirement work,	Post-retirement work,	Post-retirement work,	No post-retirement work
	all (N = 1,038)	financial need to work (N = 342)	no financial need to work (N = 615)	(N = 2,091)
Physical workload				
Work was physically too demanding (mean, 95% CI)	2.01 (1.93-2.09)	2.34 (2.19-2.49)	1.81 (1.72-1.90)	1.99 (1.94-2.05)
Psychosocial working conditions				
Job control (mean, 95% CI)	3.73 (3.65-3.80)	3.57 (3.43-3.71)	3.84 (3.75-3.93)	3.56 (3.51-3.61)
Job demands (mean, 95% CI)	2.66 (2.60-2.73)	2.79 (2.67-2.91)	2.57 (2.49-2.66)	2.97 (2.92-3.02)
Social support (mean, 95% CI)	3.58 (3.51-3.65)	3.43 (3.30-3.56)	3.66 (3.57-3.75)	3.29 (3.23-3.34)
Work arrangements				
Shift work	18.9%	22.7%	15.3%	15.2%
Part-time work	12.8%	16.3%	10.1%	7.2%
Temporary work contract	9.2%	11.7%	8.5%	3.8%
Occupational position				
Managers and professionals	32.8%	19.6%	42.0%	28.2%
Lower grade non-manual	25.0%	26.8%	23.3%	33.5%
Skilled manual workers	36.4%	43.7%	30.8%	32.0%
Elementary workers	5.8%	9.9%	3.8%	6.3%
Length of working career, years				
0-35	14.5%	21.6%	10.4%	13.1%
35-45	73.1%	64.6%	78.4%	71.9%
45+	12.4%	13.7%	11.2%	15.0%
Health and work ability				
Self-rated health				
Good	82.2%	73.3%	87.4%	80.2%
Moderate	15.7%	23.2%	11.6%	15.4%
Poor	2.1%	3.6%	1.0%	4.4%
Work ability				
0-5	4.8%	5.3%	3.4%	11.1%
6-7	21.1%	25.6%	18.6%	32.1%
8-9	65.7%	60.9%	69.1%	51.8%
10	8.4%	8.2%	9.0%	5.1%
Age in 2022, (mean, 95% CI)	66.77 (66.67-66.87)	66.68 (66.51-66.84)	66.75 (66.62-66.87)	66.30 (66.25-66.36)
Gender				
Female	59.5%	69.3%	52.9%	62.6%
Men	40.5%	30.7%	47.2%	37.5%

The results also revealed differences in pre-retirement psychosocial working conditions between working and non-working retirees. Working retirees reported greater job control (mean = 3.73; 95% CI 3.65–3.80), lower job demands (mean = 2.66; 95% CI 2.60–2.73), and higher social support (mean = 3.58; 95% CI 3.51–3.65) than those who had not worked in retirement. These factors also showed higher scores among those who worked for non-financial reasons than those who worked for financial reasons.

Furthermore, working retirees were more likely to have worked before retirement in shift work (19%), or part-time work (13%) and to have had temporary work contracts (9%) compared to those who had not worked in retirement. Such work arrangements were also more common among those who reported working for financial reasons than among those who worked for non-financial reasons.

The results also indicated that individuals who worked in retirement were often managers and professionals (33%) or skilled manual workers (36%). Those who worked in retirement for non-financial reasons were mostly managers or professionals (42%), while nearly half (44%) of those who worked for financial reasons were skilled manual workers.

Length of working career differed very little between retirees who worked in retirement and those who did not. However, 22 per cent of those who worked for financial reasons had had short careers (0–35 years) compared to only 10 per cent of those who worked for other reasons.

A relatively large number of respondents felt their health was good. Among working retirees, 82 per cent rated their health as good, compared to 80 per cent of non-working retirees. While 87 per cent of those working for other than financial reasons rated their health as good, the corresponding figure was somewhat lower, 73 per cent for those working for financial reasons. Working retirees also rated their work ability as higher than non-working retirees.

The average age of working retirees was 66.8 years, while the average age of non-working retirees was 66.5 years. Men accounted for 40 per cent of those working in retirement and for 38 per cent of those not working. Women worked for financial reasons more often than men (Table 1).

Table 2 shows the results of a logistic regression analysis on post-retirement work. All work-related factors except for physical workload were linked to post-retirement employment in models that consider their effect independently (crude odds ratios). In the adjusted model, an association was observed between high physical workload and working in retirement (OR=1.13; 95% CI 1.04–1.22). High job demands (OR=0.84; 95% 0.77–0.92) were linked to lower odds and high social support (OR=1.18; 95% CI 1.12–1.81) to higher odds of working in retirement. Furthermore, post-retirement work was more common among those who had worked in shifts (OR=1.42; 95% CI 1.12–1.81), who had done part-time work (OR=1.77; 95% CI 1.31–2.37), and who had worked temporarily (OR=2.38; 95% CI 1.64–3.44) before retirement than among those who had not done this type of work. In addition, we observed occupational class differences in post-retirement employment. Working in retirement was more common among managers and professionals (OR=1.46; 95% CI 1.17–1.81) and skilled manual workers (OR=1.29; 95% CI 1.03–1.63) compared to low-grade non-manual workers. Those with medium-length careers (35−45 years) (OR=1.36; 95% CI 1.06–1.76) worked in retirement more often than those with long (45 + years) careers.

**Table 2:** Logistic regression analysis (crude and adjusted ORs with their 95% CIs) for post-retirement work.

		Crude OR	Adjusted OR*)
		(95% CI)	(95% CI)
Physical workload	Work was physically too demanding	1.02 (0.96-1.08)	1.13 (1.04-1.22)
Psychosocial working conditions	Job control	1.12 (1.05-1.19)	0.98 (0.90-1.06)
	Job demands	0.77 (0.72-0.83)	0.84 (0.77-0.92)
	Social support	1.22 (1.15-1.31)	1.18 (1.12-1.81)
Work arrangements	Shift work	1.30 (1.07-1.59)	1.42 (1.12-1.81)
	No shift work	ref.	ref.
	Part-time work	1.88 (1.46-2.43)	1.77 (1.31-2.37)
	Full-time work	ref.	ref.
	Temporary work	2.63(1.90-3.63)	2.38 (1.64-3.44)
	Permanent employment contract	ref.	ref.
Occupational class	Managers and professionals	1.60 (1.29-1.91)	1.46 (1.17-1.81)
	Lower grade non-manual workers	ref.	ref.
	Skilled manual workers	1.54 (1.27-1.88)	1.29 (1.03-1.63)
	Elementary workers	1.24 (0.88-1.75)	0.92 (0.62-1.37)
Length of working career	0-35 years	1.34 (1.00-1.79)	1.23 (0.89-1.72)
	35-45 years	1.21 (0.97-1.52)	1.36 (1.06-1.76)
	45 + years	ref.	ref.

*) All work-related factors adjusted with health, work ability, age, and gender

In addition, we explored how pre-retirement work-related factors were linked to working in retirement for financial reasons and working for non-financial reasons. Multinomial logistic regression analysis showed odd ratios and their 95% confidence intervals for these two groups, with those who had not worked in retirement serving as the reference group (Table 3). Adjusted odds ratios indicated that a higher physical workload (OR= 1.22; 95% CI 1.09–1.37), lower job demands (OR=0.87; 95% CI 0.77–1.00), part-time work (OR=1.94; 1.31–2.88), and a temporary work contract (OR=2.44; 95% CI 1.50–3.95) were linked to working in retirement for financial reasons. Furthermore, skilled manual workers (OR=1.46; 95% CI 1.03–2.05) worked more often for financial reasons compared to lower grade non-manual workers. Career length was not statistically significantly associated with working in retirement for financial reasons in the adjusted model.

**Table 3:** Multinomial logistic regression analysis (crude and adjusted ORs with their 95% CIs) for post-retirement work among those who worked for financial reasons and for other than financial reasons.

		Post-retirement work, financial need to work		Post-retirement work, no financial need to work	
		Crude OR (95% CI)	Adjusted OR (95% CI)	Crude OR (95% CI)	Adjusted OR (95% CI)
Physical workload	Work was physically too demanding	1.22 (1.11-1.32)	1.22 (1.09-1.37)	0.89 (0.83-0.97)	1.07 (0.97-1.18)
Psychosocial working conditions	Job control	1.01 (0.92-1.11)	1.04 (0.92-1.17)	1.21 (1.11-1.31)	0.96 (0.87-1.05)
	Job demands	0.86 (0.77-0.96)	0.87 (0.77-1.00)	0.72 (0.66-0.78)	0.82 (0.74-0.90)
	Social support	1.09 (0.99-1.21)	1.04 (0.93-1.17)	1.32 (1.21-1.43)	1.24 (1.13-1.37)
Work arrangements	Shift work	1.62 (1.21-2.16)	1.25 (0.89-1.77)	1.01 (0.79-1.30)	1.39 (1.03-1.88)
	No shift work	ref.	ref.	ref.	ref.
	Part-time work	2.55 (1.81-3.60)	1.94 (1.31-2.88)	1.44 (1.04-1.98)	1.43 (0.98-2.07)
	Full-time work	ref.	ref.	ref.	ref.
	Temporary work	3.48 (2.29-5.30)	2.44 (1.50-3.95)	2.48 (1.71-3.62)	2.86 (1.86-4.41)
	Permanent employment contract	ref.	ref.	ref.	ref.
Occupational class	Managers and professionals	0.87 (0.61-1.22)	0.89 (0.62-1.28)	2.14 (1.69-2.71)	1.84 (1.43-2.38)
	Lower grade non-manual workers	ref.	ref.	ref.	ref.
	Skilled manual workers	1.72 (1.29-2.30)	1.46 (1.03-2.05)	1.40 (1.10-1.79)	1.19 (0.89-1.58)
	Elementary workers	2.00 (1.28-3.12)	1.24 (0.74-2.09)	0.86(0.53-1.39)	0.77 (0.45-1.30)
Length of working career	0-35 years	1.74 (1.15-2.62)	1.50 (0.94-2.37)	1.12 (0.76-1.65)	1.06 (0.69-1.61)
	35-45 years	0.94 (0.66-1.32)	1.13 (0.78-1.65)	1.48 (1.11-1.98)	1.52 (1.11-2.08)
	45 + years	ref.	ref.	ref.	ref.

Correspondingly, the adjusted odds ratios showed that lower job demands (OR=0.82; 95% CI 0.74–0.90), higher social support (OR=1.24; 95% CI 1.13–1.37), having worked in shift work (OR=1.39; 95% CI 1.03–1.88), and temporary work contract (2.86; 95% CI 1.86–4.41) were associated with working in retirement for other than financial reasons. Furthermore, managers and professionals (1.84; 95% CI 1.43–2.38) worked in retirement for non-financial reasons more often than lower grade non-manual workers. Retirees with medium-length careers (OR=1.52; 95% CI 1.11–2.08) worked in retirement more often than those with long careers.

## Discussion

This study examined the relationship between work-related factors in late working life and continuing to work after retiring among recently retired old-age pensioners in Finland. Our analysis covered a wide range of pre-retirement work characteristics, including physical workload, psychosocial working conditions, working arrangements, occupational position, and length of working career. We explored whether these associations varied by individuals’ motives for post-retirement employment, distinguishing between financial and non-financial reasons for continuing to work. Overall, the results suggest that although several work-related factors are linked to post-retirement employment, their importance varies somewhat depending on whether work is driven by financial or non-financial reasons.

Our results showed a relationship between favourable psychosocial working conditions and working in retirement. Post-retirement employment is facilitated by manageable workloads, minimal changes in job descriptions or skill requirements, and adequate recognition and support for older workers. These findings align with previous research suggesting that high job demands are linked to earlier retirement and withdrawal from the workforce [8], whereas favourable psychosocial conditions are linked to extended working lives [10–12,28]. In our study, we found that social support was associated with working for non-financial reasons, whereas no apparent association was observed among individuals who worked in retirement for financial reasons. From a continuity theory perspective, working in retirement may reflect a desire to maintain the social roles, identity, and daily structure derived from work. Favourable psychosocial conditions in pre-retirement employment may help sustain these aspects. For those motivated by financial reasons, continued employment seems to depend less on positive work experiences. This suggests that financial incentives may supersede psychosocial factors.

Relatively few retired people felt that their pre-retirement job was too physically demanding.  However, the perception of excessive physical demands at work was associated with an increased likelihood of working in retirement in models that controlled for other work-related factors, self-rated health, and ability to work. Furthermore, this association differed by motivation for working in retirement. Those working for financial reasons were more likely to report excessive physical demands in their pre-retirement job than those working for non-financial reasons. These findings emphasise the importance of distinguishing between different motives for post-retirement employment when interpreting the role of physical workload, since similar employment outcomes may reflect fundamentally different underlying mechanisms.

Non-standard working arrangements prior to retirement, such as shift work, part-time work, and temporary employment, were relatively uncommon among recently retired old-age pensioners. However, these arrangements were positively associated with working in retirement. This suggests that individuals with prior experience in flexible or non-standard employment may find it easier to continue working alongside their pension. Previous studies [7,24] have produced similar evidence. Experience of reduced working hours, variable schedules, or less permanent employment relationships may lower the threshold for engaging in post-retirement employment as such arrangements will be more familiar. From a theoretical perspective, these findings align with the concept of bridge employment, which refers to paid work undertaken after retiring from a career job, typically in more flexible or transitional forms. Prior experience of part-time, shift, or temporary work may facilitate this transition by enabling continuity in employment practices while allowing individuals to adjust their work participation according to their changing capacities and preferences.

Our findings show that post-retirement employment varies by occupational position. Managers and professionals are more likely to continue to work after retirement than lower-grade non-manual workers. This finding is consistent with earlier research linking higher socioeconomic status to continued employment [6,29–32]. Those in higher occupational positions typically perform less physically demanding work and have greater autonomy and control over their tasks, which may facilitate continued employment in later life. In addition, managers and professionals are more likely to work in retirement, as they often find their work meaningful and enjoyable [6,28]. For many of them, continued employment helps maintain a sense of professional identity and social status, underscoring the importance of non-economic motivations for working after retirement. In contrast, individuals in lower occupational positions are more likely to engage in post-retirement work for financial reasons [6,28]. At the same time, continuing to work after retirement may pose greater challenges for individuals with lower socioeconomic status, as they tend to experience poorer health and greater physical limitations [46,47]. Their careers often involve physically demanding tasks that increase the risk of illness and reduced functional capacity in later life [46]. These health inequalities and differences in working conditions, which accumulate over the life course, may be reflected in occupational differences in post-retirement employment.

Our results showed that retirees with very long careers (over 45 years) worked in retirement less often. One possible explanation is that retirees with long careers may feel they have worked enough and therefore no longer need to work [6]. There may also be underlying financial reasons, as shorter careers usually imply lower levels of pension accrual. Previous studies [33,34] have found that shorter working careers are often associated with a financial need to work, especially among women with career breaks and those with low income levels. Our results on the motives for post-retirement employment support this evidence, showing that retirees with shorter careers are more often motivated to work in retirement for financial reasons than those with longer careers, even though in the adjusted model this association was no longer statistically significant.

Current demographic trends in Finland pose significant long-term challenges for the labour market and pension system, as the working-age population is shrinking while the number of retirees is growing. Encouraging post-retirement employment can extend working lives, strengthen retirees’ financial security and wellbeing, and benefit society by increasing the labour supply, tax revenues, knowledge retention, and intergenerational interaction while promoting a more inclusive work culture. The findings of this study highlight the importance of working conditions and their role in shaping opportunities for post-retirement employment. Policies aimed at promoting post-retirement employment should therefore focus not only on creating retirement incentives but also on improving working conditions during the final years of regular working life. Manageable job demands, supportive work environments, and recognition of older workers’ skills and contribution appear to be particularly important in enabling individuals to continue working after retirement in meaningful work tasks. Furthermore, the association between flexible working opportunities and continued labour market participation should not be overlooked. Post-retirement employment policies need to account for the fact that different work-related factors have different importance depending on individuals’ motives for continuing to work after retirement.

### Methodological considerations

We had a reliable and representative survey dataset on recently retired old-age pensioners, and the survey yielded an excellent response rate (68%). In addition, the non-response rate for individual questions was very low. The survey included several questions on pre-retirement working arrangements and conditions. Job demands and social support were assessed using multiple items, whereas job control and physical workload were measured with only a single item each. Therefore, we may not have been able to fully capture these latter aspects. The use of multiple items for job control and physical workload would likely have yielded more reliable assessments. In addition, the survey collected information on shift work as well as part-time and temporary employment prior to retirement. All measures were based on retrospective self-reports of pre-retirement working conditions and may therefore be subject to recall and rationalization biases. Furthermore, the observed associations should be interpreted with caution and are not indicative of causal relationships. A major strength of the study lies in the linkage of survey data with reliable register-based information on the length of working careers and occupational class.

Our data represent individuals who retired from paid employment with an old-age pension between 2019 and 2021. The data do not include individuals who retired from full-time self-employment, although those who were self-employed alongside paid employment were included. The data also excludes individuals who were unemployed, work-disabled or otherwise inactive prior to receiving an old-age pension. Consequently, recently retired individuals from paid employment are a relevant group for continuing to work after retirement. These individuals are relatively healthy, capable of working, and accustomed to working life. They also possess skills that they may wish to continue using in paid work after retirement.

### Conclusions

This study demonstrates that post-retirement employment is linked to pre-retirement working conditions and occupational position. These associations differed according to individuals’ motives for continuing to work. Favourable psychosocial working conditions and higher occupational status were particularly important for individuals working in retirement for non-financial reasons. These findings suggest that meaningful work, social interaction, and supportive environments play a key role in sustaining engagement in paid work beyond retirement. Conversely, lower occupational status and physically demanding work were associated with working in retirement for financial reasons. This indicates that for some, continued employment may reflect economic necessity rather than favourable work experiences.

Efforts to promote extended working lives should address work-related factors earlier in the life course, particularly by improving working conditions before reaching retirement age. Furthermore, since the importance of these factors varies depending on the reasons for working, policy approaches should move beyond a one-size-fits-all perspective and account for such structural differences, as failure to do so may risk exacerbating social inequalities among older populations.
